# Tracking cellular therapies to optimize homing against liver metastases

**DOI:** 10.3389/fimmu.2025.1611861

**Published:** 2025-06-25

**Authors:** Megan C. Purl, Alexandria Shick, Robert J. Canter, Sean J. Judge

**Affiliations:** ^1^ University of California Davis Health, Division of Surgical Oncology, Department of Surgery, Sacramento, CA, United States; ^2^ Comprehensive Cancer Center, University of California, Davis, Sacramento, CA, United States

**Keywords:** cancer immunotherapy, cellular therapy, immune cell tracking, chimeric antigen receptor, liver metastases, regional therapy

## Abstract

Novel cellular therapies have shown practice changing results in a range of hematologic malignancies, though success against solid tumors has been limited. Key factors limiting success of these therapies against solid tumors are homing to the site(s) of disease, engraftment, maintenance of function, and persistence. The inhospitable tumor microenvironment appears to provide barriers at every step of this process. The liver, a unique organ with diverse immunoregulatory functions, is a common site for metastatic disease from solid cancers of the gastrointestinal (GI) tract. Although the complex interplay between hepatocytes, circulatory and tissue resident immune cells, and the enterohepatic circulation has been investigated for some time, many unanswered questions about the immunobiology of the liver remain. More so, novel imaging techniques provide unparalleled insight into these interactions and shed light on these complex processes that can lead to an improved understanding of the tumor microenvironment in the liver and opportunities for improving homing of cellular therapy against liver tumors. In this review, we will provide a focused assessment of this burgeoning field and focus on the emerging tools for studying homing of these therapies and how they may be enhanced to better treat liver metastases.

## 1 Introduction

The use of autologous chimeric antigen receptor (CAR) T cells has revolutionized the treatment of select hematologic malignancies. The success of CAR T therapy in these B-cell malignancies has sparked investigation into their use in solid tumors. Among gastrointestinal tumors, CAR T cells targeting claudin 18.2 has arisen as one of the most promising therapies against advanced or metastatic disease. Recent long-term results from a Phase I/II clinical trial investigating CAR T cells against claudin 18.2 for advanced GI tumors showed a 39% overall response rate among the 98 patients treated, though there were different cohorts undergoing different treatment sequencing and immunotherapy adjuncts. However, it is notable that the investigators detected significantly worse outcomes in patients with liver metastases (median PFS 3.9 months versus 7.1 months) (1). This observation may highlight unique features of the liver and show how CAR cell therapy may function differently within this organ. Several clinical trials are underway (**Table 1**) aiming to treat advanced GI malignancies with cellular therapy and with different augmentation strategies.

**Table 1:** Ongoing clinical trials investigating cellular therapies for advanced gastrointestinal malignancies.

Cell therapies for advanced GI malignancies		
NCT Number		Study Title
Systemic CAR T	NCT05239143	P-MUC1C-ALLO1 Allogeneic CAR-T Cells in the Treatment of Subjects with Advanced or Metastatic Solid Tumors
	NCT05583201	NKG2D/CLDN18.2 CAR-T(KD-496) in the Treatment of Advanced NKG2DL+/CLDN18.2+ Solid Tumors
	NCT05089266	Study of αPD1-MSLN-CAR T Cells to Evaluate the Safety, Tolerability, and Effectiveness for Patients With MSLN-positive Advanced Solid Tumors
	NCT06912152	MT027 in Patients With Advanced Peritoneal Malignancies or Abdominal Metastatic Solid Tumors
	NCT05538195	Safety and Efficacy of CEA-targeted CAR-T for CEA-positive Advanced Malignant Solid Tumors
	NCT03874897	Chimeric Antigen Receptor T Cells Targeting claudin18.2 in Solid Tumors
	NCT05605197	U87 CART in Treatment of Advanced Solid Tumor
	NCT05947487	CD70 Targeted CAR-T Cells in CD70 Positive Advanced/Metastatic Solid Tumors
	NCT05120271	BOXR1030 T Cells in Subjects With Advanced GPC3-Positive Solid Tumors
	NCT06937567	CDH17 CAR-T Therapy in Advanced Malignant Solid Tumors
Regional CAR T	NCT02850536	CAR-T Hepatic Artery Infusions or Pancreatic Venous Infusions for CEA-Expressing Liver Metastases or Pancreas Cancer
	NCT02862704	A Study of MG7 Redirected Autologous T Cells for Advanced MG7 Positive Liver Metastases(MG7-CART)
	NCT03370198	Hepatic Transarterial Administrations of NKR-2 in Patients With Unresectable Liver Metastases From Colorectal Cancer
	NCT03818165	Phase 1b Study of CAR2Anti-CEA CAR-T Cell Hepatic Infusions for Pancreatic Carcinoma Patients With CEA+ Liver Metastases
	NCT04037241	Study of Anti-CEA CAR-T + Chemotherapy VS Chemotherapy Alone in Patients With CEA+Pancreatic Cancer & Liver Metastases
	NCT04952272	Intratumor CpG-ODN Injection Boosters Immune Killing Against in Situ Tumor Antigen Release for Advanced Solid Tumors
	NCT02416466	CAR-T Hepatic Artery Infusions and Sir-Spheres for Liver Metastases
Systemic CAR T + cytokines	NCT02498912	Cyclophosphamide Followed by Intravenous and Intraperitoneal Infusion of Autologous T Cells Genetically Engineered to Secrete IL-12 and to Target the MUC16ecto Antigen in Patients With Recurrent MUC16ecto+ Solid Tumors
	NCT03932565	Interventional Therapy Sequential With the Fourth-generation CAR-T Targeting Nectin4/FAP for Malignant Solid Tumors
	NCT04377932	Interleukin-15 Armored Glypican 3-specific Chimeric Antigen Receptor Expressed in T Cells for Pediatric Solid Tumors
	NCT04715191	Interleukin-15 and -21 Armored Glypican-3-specific Chimeric Antigen Receptor Expressed in T Cells for Pediatric Solid Tumors
	NCT05035407	T Cell Receptor Gene Therapy Targeting KK-LC-1 for Gastric, Breast, Cervical, Lung and Other KK-LC-1 Positive Epithelial Cancers
	NCT05103631	Interleukin-15 Armored Glypican 3-specific Chimeric Antigen Receptor Expressed in Autologous T Cells for Solid Tumors
	NCT05393986	Claudin18.2-redirected Chimeric Antigen Receptor T Cells With Co-expression of Cytokines in Solid Tumors
	NCT06198296	Immunotherapy for Adults with GPC3-Positive Solid Tumors Using IL-15 and IL-21 Armored GPC3-CAR T Cells
	NCT05605197	U87 CART in Treatment of Advanced Solid Tumor
Systemic ACT + cytokines	NCT01868490	The Adoptive Immunotherapy for Solid Tumors Using Modified Autologous CIK Cells
	NCT01914263	Safety Study of Cord Blood-derived Cytokine-induced Killer Cells in Patients With Solid Tumor After Radical Resection
	NCT02757391	CD8+ T Cell Therapy and Pembrolizumab in Treating Patients With Metastatic Gastrointestinal Tumors
	NCT03610490	Autologous Tumor Infiltrating Lymphocytes MDA-TIL in Treating Patients With Recurrent or Refractory Ovarian Cancer, Colorectal Cancer, or Pancreatic Ductal Adenocarcinoma
	NCT06626256	STIL101 for Injection for the Treatment of Locally Advanced, Metastatic or Unresectable Pancreatic Cancer, Colorectal Cancer, Renal Cell Cancer, Cervical Cancer and Melanoma
Systemic ACT + RT	NCT04765462	Allogeneic γδT T Cell Therapy for the Treatment of Solid Tumors
	NCT03992326	Adoptive Transfer Of Autologous Tumor-Infiltrating Lymphocytes in Solid Tumors
	NCT06069570	Safety Study for a Gamma Delta T Cell Product Used With Low Dose Radiotherapy in Patients With Locally Advanced or Metastatic NSCLC or Solid Tumors With Bone Metastases
Regional CAR T + RT	NCT02416466	CAR-T Hepatic Artery Infusions and Sir-Spheres for Liver Metastases

GI, gastrointestinal; CAR T, chimeric antigen receptor T cells; ACT, adoptive cell transfer; RT, radiation therapy.

The liver contains a diverse repertoire of immune and non-immune cells including liver sinusoid endothelial cells (LSEC), hepatic stellate cells, hepatocytes, Kupffer cells (KCs), monocyte-derived macrophages, myeloid-derived suppressor cells (MDSCs), natural killer (NK) cells, neutrophils, and T lymphocytes. These cell populations have broad and often opposing roles within the context of liver metastases. For example, Kupffer cells and monocyte-derived macrophages can demonstrate anti-tumor effects through direct phagocytosis of cancer cells, the production and pro-inflammatory cytokines such as TNF-a and the recruitment of cytotoxic T lymphocytes and NK cells (2). However, these same cells also demonstrate pro-tumorigenic actions. KCs have been shown to participate in the pre-metastatic niche formation (3), production of various cytokines and tumorigenic growth factors including IL-6, hepatocyte growth factor (HGF), vascular endothelial growth factor (VEGF), and matrix metalloproteinases (MMP) (2, 4), while monocyte-derived macrophages can be polarized to M2 where they also secrete growth factors such as VEGF (5), and induce regulatory T cells which can inhibit cytotoxic T cells via IL-10 and TGF-b (6). This diverse and highly complex environment is further complicated in the context of cellular therapies such as CAR T, where the interplay between these therapeutic cells and the liver milieu are less understood. Given the limited ability of CAR T cell therapy to successfully treat solid tumors metastatic to the liver, further insights into the unique nature of liver metastases and how CAR cells may, or may not, be homing and functioning within the tumor-bearing liver are needed.

## 2 Liver metastases from gastrointestinal cancers

Malignancies of the GI tract represent some of the most common cancers in the United States and globally (7, 8). These include malignancies of the colon, esophagus, pancreas, rectum, and stomach with the most common histology being adenocarcinoma. Similar across these disease sites is the prevalent rate of liver metastases from the primary tumor (9). Observational data from the SEER database indicate that about 5% of patients present with synchronous liver metastases at the time of their diagnosis. Malignancies with the highest rate of synchronous liver metastases are cancers of the pancreas (36%), colon and rectum (27%), small bowel (15%), stomach (14%), and esophagus (14%) (10). Among all age groups, the highest incidence of liver metastases at diagnosis was among patients with cancers of the pancreas, colon and rectum. Better treatment of liver metastases is therefore a critical and urgent unmet need.

The current approach to treating liver metastases from GI cancers is mainly based on the primary cancer site. Treatment may include systemic therapy, radiation, or resection, though this depends on the extent of disease. In colorectal cancer, management of isolated liver metastases focuses on resection or other regional therapies such as radiofrequency ablation, arterial directed therapies, and selective internal radiation. Results from a large, multi-centered randomized clinical trial showed no improvement in overall survival (OS) when adding systemic chemotherapy to resection compared to resection alone in the management of colorectal liver metastases (median OS 61.3 months vs. 54.3 months) (11). This is in contrast to pancreas cancer where systemic chemotherapy is standard for patients with metastatic disease, and small case series investigating the role of simultaneous pancreas and liver resection in well-selected patients with low volume liver metastases identified no difference in OS compared to patients receiving palliative bypass without curative surgery and adjuvant chemotherapy (12). Apart from systemic therapy, and unique to the management of liver metastases, is the role of liver-directed, regional therapies. This was recently highlighted in the management of pancreatic cancer with limited liver or lung metastases where patients were randomized to chemotherapy or chemotherapy with targeted radiation to the metastatic sites and investigators detected improved PFS in the group receiving metastasis-directed RT (13). More so, these investigators analyzed patient peripheral blood and identified changes in circulating immune cells and cytokines that were positively associated with better PFS, highlighting the role of the immune system as a putative mediator of the anti-tumor effects following regional RT.

## 3 Liver homing of systemic cellular therapies

In the initial report of the clinical efficacy of the autologous CD19 CAR T therapy, CTL019 (tisaganleucel), for CD19+ lymphoid malignancies (14), responders had a median peak expansion of the cell therapy product at 8 days and 14/16 responders had consistently detectable levels of CTL019 DNA in their peripheral blood between 6 and 24 months after infusion. While peripheral blood is frequently the source for measuring CAR cell persistence in hematologic malignancies, peripheral blood may not be the most representative source for CAR cell persistence and function in the setting of solid tumors. Detecting the presence of CAR cells within a tumor-bearing solid organ is a challenging clinical issue and is either completed using a biopsy or requires investigation at autopsy. An earlier Phase I safety trial evaluating a second-generation CD19 CAR T cell therapy against B-cell leukemias investigated CAR T cell presence in other tissue sites following patient death. In a patient with CLL, researchers evaluated tumor contained within the lymph nodes, liver and bone marrow and identified the presence of these modified cells 44 hours after CAR T infusion (15). These data suggest that CAR T cells can home to sites of disease when given systemically. However, although CAR T cells can traffic to solid organs and tissues, it is not clear what effect infiltration into solid organs and tumors has on CAR T cell function. While there is an association between persistence of the cell therapy product and improved long term clinical outcomes (16), this does not appear necessary to prevent disease relapse. Additionally, relapse can still occur in the presence of persisting CAR T cells. In a recent review investigating long-term outcomes after CAR T therapy for hematologic malignancies, researchers cite various examples where durable responses have occurred in patients with no long-term evidence of CAR T persistence, and the setting where disease recurs while CAR T cells remain detectable (17). Using techniques to maximize homing of the cell therapy to sites of disease may lead to increased function and persistence by placing the CAR in proximity to the intended antigen. While these data show that systemically administered CAR cells can home to the liver, the larger question is whether this represents passive circulation of the CAR T cells through a highly vascular organ, or rather an active process where the CAR T cells preferentially traffic to the liver based on antigen recognition, chemokine or cytokine gradients, or some other pro-infiltrative signal.

## 4 PET imaging for tracking cellular therapies

Positron emission tomography (PET) is a nuclear medicine imaging technique that utilizes radioactive tracers to track both typical and atypical metabolic activity. PET/CT is commonly used for staging, assessment of therapeutic responses, and surveillance for different cancer types, including GI malignancies (18). The most commonly used PET radiotracer is 2-deoxy-2-[^18^F] fluorodeoxyglucose ([^18^F]FDG) which identified tissues with elevated glycolysis by radiotracer uptake. PET imaging can also be used to track cellular therapies *in vivo*. Techniques for tracking CAR cell therapy have been driven in large part due to advances in novel PET imaging probes. These technologies have allowed researchers to evaluate the variables of time and dose as a function of CAR homing and persistence, however these studies can be limited by the short half-life of these tracers. In one such study, investigators used CD19-tPSMA CAR T cells tagged with 18-fluorinated-DCFPyL against an acute lymphoblastic leukemia (ALL) mouse model to characterize CAR T durability and persistence (19). While the CAR T cells homed to tumor and had a profound anti-tumor effect, CAR T infiltration into tumor did not correlate with the concentration of CAR T cells within the peripheral blood or bone marrow, suggesting that peripheral blood levels may not represent what’s occurring within the sites of disease. These results highlight the challenges in using peripheral blood as a surrogate in solid tumors.

Other PET-based imaging modalities are also being investigated to track cellular therapies *in vivo*. Immuno-positron emission tomography (ImmunoPET) is increasingly used in cellular therapies by combining the specificity of monoclonal antibodies with the high sensitivity and resolution of PET through utilizing radiolabeled antibodies and antibody fragments. These immunoPET probes such as the ^89^Zr-DFO anti-ICOS tracer have been used to track human CAR T cells *in vivo* (20). Other strategies include Immuno-PET/SPECT imaging which combines immunoPET with single-photon emission computed tomography (SPECT) to track CAR T cell therapies within solid tumors. This method has also been demonstrated to detect CAR T distribution, engraftment, and clearance within the setting of solid tumors (21). While these antibody-based imaging strategies have shown to be quite advantageous, the choice of antibody conjugation strategy and radionuclide half-life can determine the effectiveness of these methods and are important considerations when designing these experiments. The goal of these imaging techniques is to further enhance our understanding of cellular therapy in the setting of solid tumors and give insight into how to better improve these therapies. Various avenues have been investigated in order to improve these cellular therapies including route of administration, as well as enhancement with immunomodulatory agents such as radiation therapy and cytokines.

## 5 Regional administration to enhance liver homing

In clinical practice certain regional therapies have exploited the unique vasculature of the liver. This is most notable with the use of hepatic artery infusion therapy that utilizes high potency chemotherapeutics instilled directly into the hepatic artery to target liver metastases. Since the chemotherapy undergoes rapid first-pass metabolism in the liver, the agents can deliver targeted doses of chemotherapy to the liver before entering the systemic circulation via hepatic venous outflow as an inert chemotherapy byproduct. Several prior studies have clearly demonstrated that liver metastases are preferentially fed by the hepatic arterial system, as compared to the portal venous system. More so, pre-clinical studies have shown that higher concentrations of active chemotherapy are detected within liver tumors when the chemotherapy is administered via the hepatic artery compared to the portal vein (22). While these observations make a clear case for utilizing the hepatic arterial system for infusion of high potency chemotherapy, portal venous infusion appears to be the preferred route of administration for islet cell transplantation for the treatment and prevention of diabetes (23). While the reason for this observation remains unknown, those prior investigators posited that it may be related to relative ischemia at the end arterioles limiting islet cell survival and persistence. Nonetheless, there appears to be relevant differences between these two vascular systems in how the inflow arrives and is metabolized within the liver parenchyma, and this may be especially true when dealing with a cellular therapy.

### 5.1 Hepatic artery and portal vein delivery

Hepatic artery infusion therapy has been used for decades with favorable results (24). While this platform is most frequently utilized to deliver high-dose chemotherapy directly to liver tumors, new treatment options have arisen with the advent of novel therapies, including immunotherapy and cellular therapy. More recently, CAR T cells targeting the CEA antigen have been administered via the hepatic artery without or with systemic interleukin (IL)-2 support to target GI cancers metastatic to the liver. In a phase I trial of 8 patients, investigators showed the safety and feasibility of CAR T infusion into the hepatic arterial system for unresectable liver metastases from diverse GI malignancies (25). Investigators also collected a targeted and non-targeted liver biopsy at the time of the third CAR T infusion and evaluated the persistence of the product. The authors noted that nearly 1% of the normal liver mononuclear cells were CAR+ and 6.6% of the intratumoral mononuclear cells were CAR+, suggesting at least some preferential homing to the site of disease. Additionally, in four of the patients CAR T cells were not detectable in peripheral blood, suggesting that either the cell therapy product does not consistently enter systemic circulation, or that there is poor persistence of the cells within the periphery.

While most current CAR T or CAR NK studies are not utilizing regional therapy, prior investigators examined the effect of infusing lymphokine activated killer (LAK) cells into the liver for treating metastases from various primary sources. Using peripheral blood mononuclear cells exposed to IL-2 *in vitro*, LAK cells were generated and administered. Investigators delivered LAK cells into either the portal vein or hepatic arterial system in patients with liver metastases from melanoma (26). In a subset of patients, these LAK cells were radio-labelled and followed *in vivo* with imaging. *In vivo* distribution was monitored 24 and 120 hours after infusion and showed that over 80% of the radioactivity remained in the liver while the remainder of the radioactivity was in the spleen. This observation was noted to be similar between portal venous and hepatic arterial administration at both timepoints. The authors did not report a difference in anti-tumor effect between these two routes. While these results do not show a difference in homing between the route of regional delivery, it does support regional delivery as a technique to maximize cellular engraftment into the liver when targeting liver metastases.

### 5.2 Technique and safety of regional delivery

The dual blood supply to the liver allows administration of regional therapies via either the hepatic arterial or portal venous systems. For both routes, direct and indirect access are possible. Access to the portal vein is commonly performed via either image-guided percutaneous venous access (often trans-hepatic) or direct needle cannulation at the time of surgery. Access to the hepatic arterial system is commonly performed via selective cannulation following peripheral arterial access, image-guided percutaneous arterial access, or through a hepatic arterial infusion device placed during a surgical procedure. For percutaneous portal vein access, most experience is from trans-jugular intrahepatic portosystemic shunt (TIPS) and islet cell transplantation. In a large series from Japan investigating complications after transhepatic portal vein access, the overall complication rate was 16.5% (bleeding, pleural effusion, bile leak, liver dysfunction), highlighting that bleeding is the most common complication (27). In a more contemporary series of surgical portal vein access for islet cell transplantation following total pancreatectomy, investigators measured a post-operative portal vein thrombosis rate of 6.6% (12/183), with most resolving after anti-coagulation therapy (28). This low thrombosis rate may be related to the large size and high flow volumes of the portal vein and support the safety of direct access during surgery.

Hepatic arterial access is most often completed using selective cannulation or through placement of a hepatic arterial infusion (HAI) device. Selective cannulation is a common technique and is most often utilized for trans-arterial embolization (TAE) for primary liver tumors. In one large study of nearly 5000 hepatic artery catheterizations for TAE, the incidence of arterial dissection of the celiac or its major branches was only 1.3% (61/4791) (29). This approach appears safe, but typically only allows for single treatment sessions. HAI therapy, where a permanent catheter is placed into a major hepatic arterial branch, allowing for continuous infusions and has been used for decades. In a large single-center series evaluating complications in HAI pump placement, the overall complication rate was 22% (120/544) with hepatic arterial thrombosis having occurred in 33 patients (6% total rate) (30), Notably, this thrombosis rate is similar to the portal venous thrombosis rate in the prior study. These results highlight the overall safety of these interventions however, it is important to note that these low complication rates were reported in high-volume centers with significant institutional experience in these procedures.

## 6 Radiation and cytokines to enhance liver homing

Radiation therapy (RT) has well established benefits in the multimodality management of solid tumors. RT has also been shown to demonstrate a variety of immunomodulatory effects on tumors and is now being investigated for its ability to enhance the engraftment and function of CAR therapies (31, 32). While there are currently few clinical studies investigating the combination of RT with CAR therapies, there is good evidence demonstrating the synergistic effects and enhanced antitumor efficacy with other immunotherapies such as PD-1/PD-L1 blockade (33). RT represents an additional locoregional strategy to improve cellular therapy against solid tumors, specifically cancers of the GI tract.

It has recently been shown that RT has a variety of immunomodulatory effects including direct effects on the tumor microenvironment (34, 35). It has also been shown to improve immune cell homing into the tumor via modulation of endothelial adhesion molecules intracellular adhesion molecule 1 (ICAM-1) and vascular-cell adhesion molecule 1 (VCAM-1), facilitating increased adherence and extravasation of lymphocytes out of circulation and into the tumor (36, 37). RT has also been shown to mediate increased T cell infiltration by inducing T cell attracting chemokines such as CXCL9, CXCL11, CCL5, and CCL8 in tumor cells (38, 39). Furthermore, the combination of RT with CAR T therapy has demonstrated increased CAR T infiltration into tumors with increased efficacy in a number of preclinical studies of GI malignancies. Amit and colleagues demonstrated that proton radiation boosts efficacy of CAR T therapy against pancreatic cancer. Using an orthotopic pancreatic cancer model, investigators treated mice with RT and subsequently injected mesothelin targeted CAR T cells (40). Not only did they observe an increase of CAR T infiltration into tumors but also saw an increase in tumor mesothelin expression following RT, further augmenting CAR-mediated antitumor responses. In a similar pancreatic cancer model, investigators showed that RT in combination with CAR T therapy increased CAR T efficacy and increased antigen-negative tumor susceptibility to CAR therapy (41). Jin and colleagues exploited radiation-induced IL-8 expression from tumors by utilizing CAR T cells expressing IL-8 receptors (CXCR1 or CXCR2). This resulted in enhanced migration and persistence of the CAR T cells in the tumor and was accompanied by tumor regression in pre-clinical models of pancreatic cancer (42). These pre-clinical studies demonstrate that combining RT with CAR T could enhance CAR T trafficking and have synergistic antitumor effects. In the context of GI liver metastases, RT can be directed specifically towards the liver and represents a promising modality to enhance the efficacy of cellular therapy towards solid tumors.

Cytokines represent an additional strategy by which to enhance the homing, engraftment, function, and persistence of CAR T therapy within solid GI tumors. While cytokine administration, namely IL-2, has historically been shown to have anti-tumor activity on their own (43), they are becoming increasingly used within the context of cellular immunotherapy, either via systemic administration or encoded within the cellular product to enhance therapeutic efficacy. In the context of CAR T therapy, 4^th^ generation CARs or TRUCKs (T cell Redirected Universal Cytokine-mediated Killing) are being developed that secrete cytokines to signal in an autocrine fashion for increased efficacy and anti-tumor activity (44). Examples of cytokines currently under investigation include IL-2, IL-7, IL-12, IL-15, IL-18, IL-21, among others (45–50). There are also a number of clinical trials that are investigating cytokine-armored CAR T therapies for solid GI malignancies including IL-12, IL-15/IL-21, IL-7, CCL19 (NCT02498912, NCT06198296, NCT05035407). Allen and colleagues engineered CAR T cells with a synthetic Notch receptor which secrets IL-2 upon tumor recognition. In orthotopic PDAC models using immunocompetent mice, these authors demonstrated that the addition of the IL-2 circuit to the CAR T not only enhanced anti-tumor efficacy but specifically increased the infiltration of CAR T cells into the tumors, demonstrating the importance of cytokines in CAR T infiltration into solid tumors (46). Chemokine gradients have also been investigated to enhance the trafficking of CAR T therapies to solid tumors. Wang and colleagues developed an anti-mesothelin CAR T that co-expresses the chemokine receptor CCR2b for treatment of preclinical models of non-small-cell lung carcinoma which demonstrated superior tumor infiltration compared to the Msln-CAR alone (51). This demonstrates translational potential to liver tumors as CCR2 has been shown to be involved in multiple stages of liver pathology including tumor progression (52). Thus, cytokines represent a promising strategy for enhancing the trafficking and function of CAR T therapy to solid tumors, specifically GI malignancies. While combination of CAR T therapy with strategies such as RT and cytokines do represent great promise for enhancing the efficacy of CAR T therapy against solid tumors, it is important to acknowledge the limitations of these strategies, primarily the safety of these methods. CAR T therapy alone has well documented risks of cytokine release syndrome and neurotoxicity and when combined with RT and/or cytokines which also have individual risks of toxicities, it’s important to be cognizant of these risks and develop strategies that aim to mitigate these toxicities.

## 7 Conclusion

**Figure 1:**
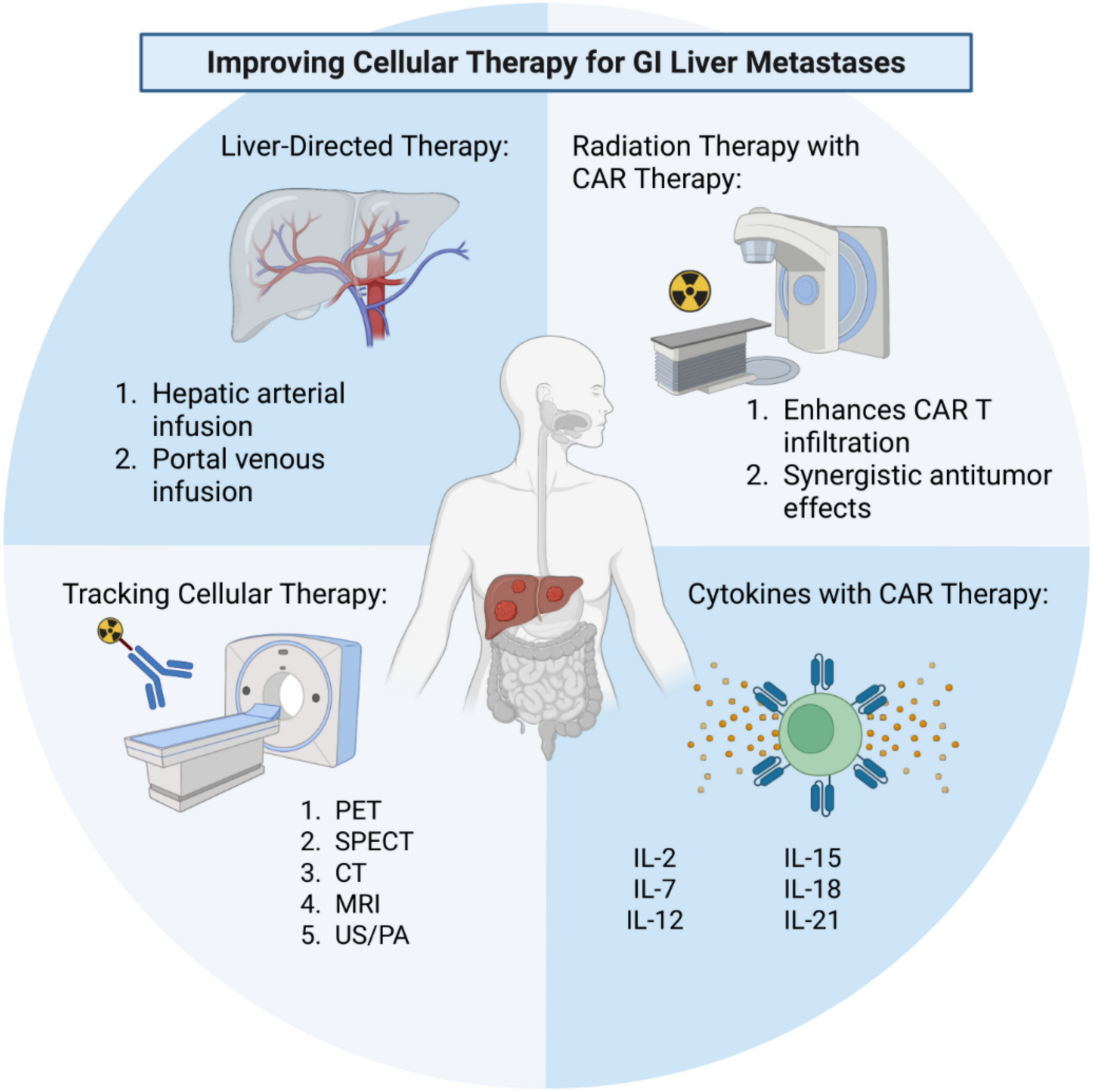
Strategies and techniques to improve cellular therapy for liver metastases of gastrointestinal (GI) origin. These include liver-directed regional therapies, combinations with external beam radiation therapy, augmenting CAR therapy with cytokines, and tracking CAR therapy using modern imaging modalities and novel probes. CAR, chimeric antigen receptor; PET, positron emission tomography; SPECT, single-photon emission computed tomography; CT, computed tomography; MRI, magnetic resonance imaging; US/PA, ultrasound/photoacoustic imaging. Created in BioRender. Purl, M. (2025) https://BioRender.com/4givi1o.

Chimeric antigen receptor therapies have revolutionized the treatment of hematological malignancies, but there is still much work to be done in optimizing these therapies for solid tumors. Several techniques are being investigated to optimize cell therapy function (**Figure 1**). Fundamental barriers to their success in solid tumors include homing, engraftment and persistence within the organ of disease. The liver is a common site of metastasis of GI malignancies and provides a unique opportunity to investigate the complex interactions between the host immune system, tumor microenvironment and cellular therapies. Novel liver-directed therapies are needed to improve cellular therapies in the context of GI liver metastases. Locoregional delivery of cellular therapies via hepatic arterial infusion or portal venous infusion represent two strategies for enhancing the homing, engraftment, and persistence of cellular therapy in the liver. While these strategies have long been tested in the application of chemotherapy, they represent a promising avenue for use in the setting of cellular therapy, specifically CAR T therapy for liver-directed treatments. Radiation therapy represents a second promising approach to enhancing the engraftment of CAR T therapy into solid tumors, specifically the liver. Studies investigating the combination of RT with CAR T therapy continue to emerge and have demonstrated promising success in the preclinical setting as a method of enhancing CAR T engraftment into solid GI malignancies. Utilizing cytokines in combination with CAR T therapy represents an additional strategy for enhancing homing and function of CAR T therapy into solid tumors. Lastly, tracking cellular therapy in real time is crucial in furthering our understanding of cellular therapy in the setting of solid tumors so that we may improve our approaches. Positron emission tomography, specifically immunoPET, has emerged as an innovative method to track CAR T therapy and has potential to provide vital information regarding the successes and failures of CAR T therapy. These tools can be utilized together to shed light on the complex interactions between host immunity, cellular therapy and the tumor microenvironment thus leading to improved cellular therapy against solid tumors.
